# The Testicular and Epididymal Expression Profile of PLCζ in Mouse and Human Does Not Support Its Role as a Sperm-Borne Oocyte Activating Factor

**DOI:** 10.1371/journal.pone.0033496

**Published:** 2012-03-12

**Authors:** Mahmoud Aarabi, Yang Yu, Wei Xu, Man Y. Tse, Stephen C. Pang, Young-Joo Yi, Peter Sutovsky, Richard Oko

**Affiliations:** 1 Department of Biomedical and Molecular Sciences, School of Medicine, Queen's University, Kingston, Ontario, Canada; 2 Department of Genetics, Reproductive Biotechnology Research Center, Avicenna Research Institute, Tehran, Iran; 3 Division of Animal Sciences, University of Missouri, Columbia, Missouri, United States of America; 4 Departments of Obstetrics, Gynaecology and Women's Health, University of Missouri, Columbia, Missouri, United States of America; McGill University, Canada

## Abstract

Phospholipase C zeta (PLCζ) is a candidate sperm-borne oocyte activating factor (SOAF) which has recently received attention as a potential biomarker of human male infertility. However, important SOAF attributes of PLCζ, including its developmental expression in mammalian spermiogenesis, its compartmentalization in sperm head perinuclear theca (PT) and its release into the ooplasm during fertilization have not been established and are addressed in this investigation. Different detergent extractions of sperm and head/tail fractions were compared for the presence of PLCζ by immunoblotting. In both human and mouse, the active isoform of PLCζ was detected in sperm fractions other than PT, where SOAF is expected to reside. Developmentally, PLCζ was incorporated as part of the acrosome during the Golgi phase of human and mouse spermiogenesis while diminishing gradually in the acrosome of elongated spermatids. Immunofluorescence localized PLCζ over the surface of the postacrosomal region of mouse and bull and head region of human spermatozoa leading us to examine its secretion in the epididymis. While previously thought to have strictly a testicular expression, PLCζ was found to be expressed and secreted by the epididymal epithelial cells explaining its presence on the sperm head surface. *In vitro* fertilization (IVF) revealed that PLCζ is no longer detectable after the acrosome reaction occurs on the surface of the zona pellucida and thus is not incorporated into the oocyte cytoplasm for activation. In summary, we show for the first time that PLCζ is compartmentalized as part of the acrosome early in human and mouse spermiogenesis and is secreted during sperm maturation in the epididymis. Most importantly, no evidence was found that PLCζ is incorporated into the detergent-resistant perinuclear theca fraction where SOAF resides.

## Introduction

Assisted Reproductive Technologies (ART) including *in vitro* fertilization (IVF) and intracytoplasmic sperm injection (ICSI) provide treatment options for infertile couples; a problem that is estimated to affect 15% of world population [1], [2]. While over 146,000 ART cycles were performed in United States in 2009, most of these cycles (63%) did not produce pregnancy [3], similar to results from European countries [4]. Failure of ART to conceive babies could be partially due to sperm inability to induce intracellular calcium release and resumption of second meiosis in the oocyte, a process called oocyte activation [5]. Oocyte activation includes the sperm-induced molecular and cellular events that occur during the transition from haploid gametes to a diploid zygote [6].

It is well established that oocyte activation is initiated after sperm delivers a testis-specific, sperm-borne oocyte activating factor (SOAF) into the ooplasm [7]. However, it is still unclear what sperm protein(s) play this crucial role. To be considered as SOAF, a candidate protein should meet specific developmental and functional criteria. Microinjection of SOAF complementary RNA (cRNA) or recombinant protein into the oocyte is expected to mimic the oocyte activation induced by ICSI. This effect includes the induction of calcium oscillations in mammalian metaphase II arrested oocytes followed by formation of secondary polar body and a female pronucleus [6]. Moreover, relevant antibodies and competitive peptides derived from a SOAF candidate should block the sperm induced oocyte activation during ICSI. SOAF should reside and remain, during early stages of fertilization, in the postacrosomal sheath [8] of sperm perinuclear theca (PAS-PT), a detergent- resistant layer surrounding sperm nuclei [9]. In order to be a PAS-PT component a SOAF candidate should originate during spermatid elongation [10]. Because of potential benefits of SOAF in the diagnosis and treatment of male infertility, it has been highly sought after. Several candidates have been considered in the past [11], [12], [13], while postacrosomal WW binding protein (PAWP) [14], [15], [16], citrate synthase [17] and phospholipase C zeta (PLCζ) [18] are presently under investigation.

PLCζ is a member of phospholipase family that was suggested as a SOAF candidate in 2002 [19]. It was demonstrated that microinjection of PLCζ cRNA or recombinant protein initiates intracellular calcium release and oocyte activation [19], [20]. In a proportion of patients with globozoospermia, where multiple defects such as absence of acrosome and PAS-PT, impaired sperm cytoskeleton and sperm inability to activate oocyte are observed [21], PLCζ was shown to be absent or defective [22], [23]. Sperm from these patients failed to activate the mouse oocyte, unless artificially activated by calcium ionophore [24]. PLCζ mRNA is present in a range of spermatogenetic phases (*i.e.* spermatocytes, round and elongating spermatids) dependent on species [19], [25], [26], [27]. However, there is no information about the developmental expression of PLCζ protein during mammalian spermatogenesis, except for equine where it is localized to the round spermatids [28]. In the spermatozoa, it has been localized to varied locations including the post acrosomal, acrosomal, equatorial and mid-piece regions [28], [29], [30], [31], [32]. In addition neither PLCζ localization during fertilization nor its non-redundancy as a SOAF has been demonstrated yet [33]. Despite past efforts, there is no evidence that PLCζ is released from sperm head PT into the oocyte cytoplasm immediately after sperm-oolemma fusion that coincides with oocyte activation.

In order to clarify whether PLCζ meets these crucial characteristic requirements of SOAF, we investigated the developmental expression of the protein within the testis and epididymis. We identified the pattern of PLCζ secretion during spermatogenesis and epididymal maturation, and followed its fate during *in vitro* fertilization. Based on our results, we suggest that PLCζ does not meet some of the important criteria to be considered as SOAF.

## Results

### Localization of PLCζ in sperm head and tail fractions

**Figure 1:**
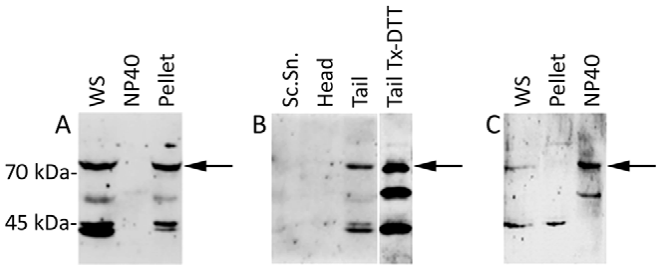
Immunoblotting of PLCζ after differential extraction of mouse and human spermatozoa. (**A**) Detergent extraction of mouse cauda epididymal spermatozoa. The ∼74 kDa band corresponding to the functional isoform of PLCζ (arrow) was detected in whole sperm (WS) and was resistant to NP40 extraction. Anti-hmPLCζ was used to detect the signal with a similar result obtained with anti-EF. (**B**) The NP40 resistant sperm fraction (*i.e.*, pellet in panel A) was sonicated and sperm heads and tails were separated through a sucrose gradient. The 74 kDa active isoform was found only in the tail fraction (arrow). Further incubation of the tail fraction with Triton x-100/DTT (Tail Tx-DTT) extracted all of the PLCζ bands, suggesting the mitochondrial sheath of the mid piece as the origin of PLCζ in the sperm tail. Anti-EF was used to detect the signal with a similar result obtained with anti- hmPLCζ. Lanes were run on the same gel but were non-contiguous. (**C**) Human sperm extraction by NP40. The functional isoform (arrow) was detergent extractable as shown in NP40 lane. Anti-hmPLCζ was used for detection. A similar result was observed with anti-EF and anti-hPLCζ. WS, Whole sperm; Sc.Sn, Sonication supernatant.

Immunoblotting of the whole mouse cauda-epididymal sperm extracts with a peptide affinity purified anti-PLCζ antibody revealed four major bands of approximately 74, 55, 45 and 43 kDa (Fig. 1A, WS). Non-ionic detergent, NP40 extracted a faint 58 kDa band while the other major bands, including the presumably functional 74 kDa PLCζ band remained in the pellet (Fig. 1A). This finding prompted us to separate the NP40 extracted sperm heads and tails by sonication and sucrose gradient centrifugation, in order to locate in which fraction the detergent insoluble PLCζ bands resided. Surprisingly, PLCζ immunoreactivity was not observed in the isolated sperm heads or sonicated supernatant, but rather in the isolated sperm tails (Fig. 1B). Incubation of the isolated tails with Triton X100-DTT completely extracted all PLCζ immunoreactive bands, indicating a mitochondrial sheath origin (Fig. 1B).

In the human spermatozoa, two distinct PLCζ–reactive bands of approximately 74- and 45- kDa were observed in whole ejaculated sperm extracts (Fig. 1C, WS). However, the 74 kDa band and most likely its breakdown product, a 55 kDa protein band, were extracted by NP40, while the detergent insoluble 45 kDa band remained in the pellet (Fig. 1C).

### Developmental expression of PLCζ during spermiogenesis by light and electron microscopy

**Figure 2:**
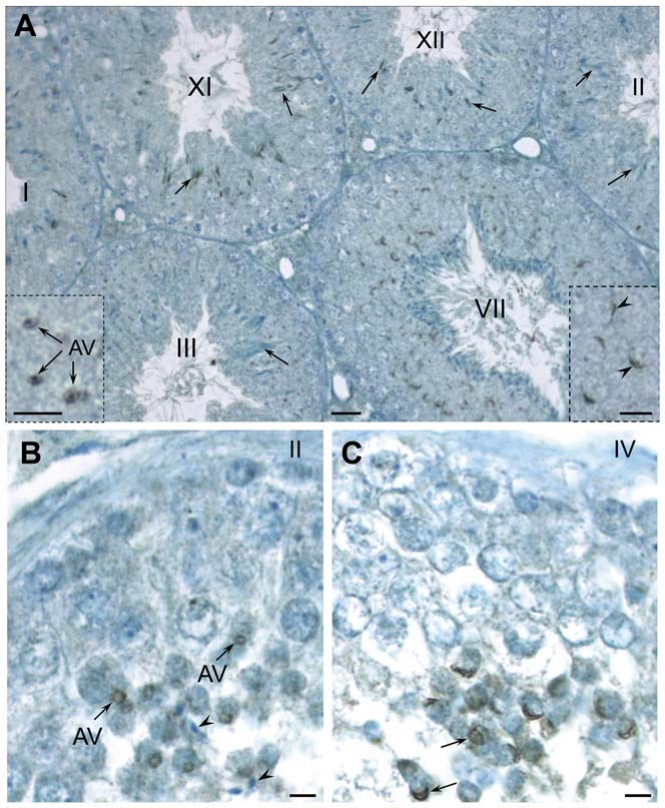
Developmental localization of PLCζ in mouse (A) and human (B, C) testes utilizing anti-hmPLCζ antibody. Similar results were obtained with anti-EF antibody in mouse and human and anti-hPLCζ in human sections. (**A**) Immunostaining originates at the beginning of acrosome formation. In stage III it appears confined to the acrosomic vesicle (AV) of step 3 spermatids as seen in detail in the inset; note that acrosomic granule is most immunoreactive. In stage VII immunostaining is intense within the acrosome, capping a portion of the nucleus (arrowheads) of the step 7 round spermatids as seen in more detail in the inset. In stages XI and XII immunostaining resides in the head of elongating spermatids in steps 11 and 12, respectively (arrows). However, by step 14 (stages II and III) the intensity of immunostaining in elongated spermatid heads has diminished significantly (arrows). (**B, C**) In human, there are six stages (I–VI) of the cycle of the seminiferous epithelium. As seen in B, PLCζ accumulates over the acrosomic vesicle (AV) of step 2 spermatids in stage II. Little immunoreactivity is found elsewhere in the epithelium and the elongated spermatids (arrowheads) appear unreactive. In step 4 spermatids (stage IV, see C), the fully formed acrosome is intensely labeled (arrows). Bars = 20 µm; Bars in insets = 5 µm.

We performed immunoproxidase staining on mouse and human paraffin embedded testicular tissues. In mouse, PLCζ was detected at the stage III of spermatogenesis in the acrosomic vesicles (AV) of step 3 round spermatids. The immunoreactivity was concentrated in the acrosomic granule, located at the center of AV (Fig. 2A). PLCζ immunostaining remained intense over the acrosomal cap in stage VII and resided in the head of elongating spermatids in stages XI and XII. Surprisingly, by step 14 spermatids (stages II and III of spermatogenesis), the intensity of immunostaining associated with elongated spermatid nuclei diminished substantially. Little or no PLCζ immunostaining was detected in spermatids at the end of spermiogenesis, except in opportune sections through the sperm tails (data not shown).

In human testicular tissues, immunostaining localized PLCζ to the AVs of step 2 spermatids in stage II of the six stage cycle of the human seminiferous epithelium (Fig. 2B). In step 4 spermatids (stage IV) immunostaining was concentrated over the fully formed acrosome, covering the apical half of the spermatid nucleus. Importantly, little immunoreactivity was found elsewhere in the epithelium and elongated spermatids (Fig. 2C). In both species, saturation of antibodies with oligopeptides used to raise the immune serum resulted in elimination of immunostaining over the round spermatids of seminiferous tubule sections in similar stages of development, confirming the specificity of reactions (Fig. S1).

**Figure 3:**
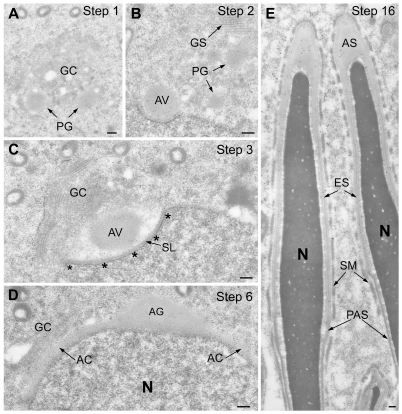
LR-White embedded mouse testicular sections immunogold labeled with affinity purified anti-hmPLCζ antibody. Similar results were obtained by anti-EF. (**A**) Section through portion of Step 1 spermatid showing Golgi complex (GC), containing several proacrosomic granules (PG) that are immunoreactive for PLCζ. (**B**) By step 2, several immunoreactive proacrosomic granules have fused with each other forming the acrosomic vesicle (AV), which attaches to the spermatid nucleus (N). (**C**) Section through a portion of step 3 spermatid showing the immunogold labeled AV docked onto the nucleus with the subacrosomal layer (SL) intervening. Underlying the nuclear envelope in the region of the forming acrosome is the electron dense nuclear lamina (outlined by asterisks). (**D**) As acrosome capping proceeds over the apical half of the nucleus in step 6 spermatids a high concentration of immunogold labeling is evident in the expanding acrosomal collar (AC) coincident with a loss of labelling in the acrosomic granule. (**E**) Step 16 spermatids, almost fully formed, show a diminution of labeling in the acrosome and no labeling is detected in the perinuclear theca. AS, apical segment of acrosome; ES, equatorial segment of acrosome; GC, Golgi complex; GS, Golgi saccules; N, nucleus; PAS, postacrosomal sheath; SM, Sertoli cell mantle. Bars = 0.2 µm.

To confirm and clarify immunoperoxidase findings, anti-PLCζ immunogold labelling was performed on mouse testicular sections prepared for electron microscopy. PLCζ was detected in the proacrosomic granules or vesicles within the Golgi complex of steps 1 and 2 round spermatids (Fig. 3A, B). Through steps 2–4, the PLCζ-containing granules fused together to from the AV, which docked onto the spermatid nucleus (Fig. 3C). During the acrosome capping process in steps 5–7, the immunogold labeling intensity shifted from the acrosomic granule to the acrosomal collar, suggesting redistribution of PLCζ from the acrosomic granule (Fig. 3D). Throughout the last steps of spermiogenesis there was a noticeable diminution in the intensity of acrosome labelling (Fig. 3E).

### Immunofluorescence localization of sperm PLCζ

**Figure 4:**
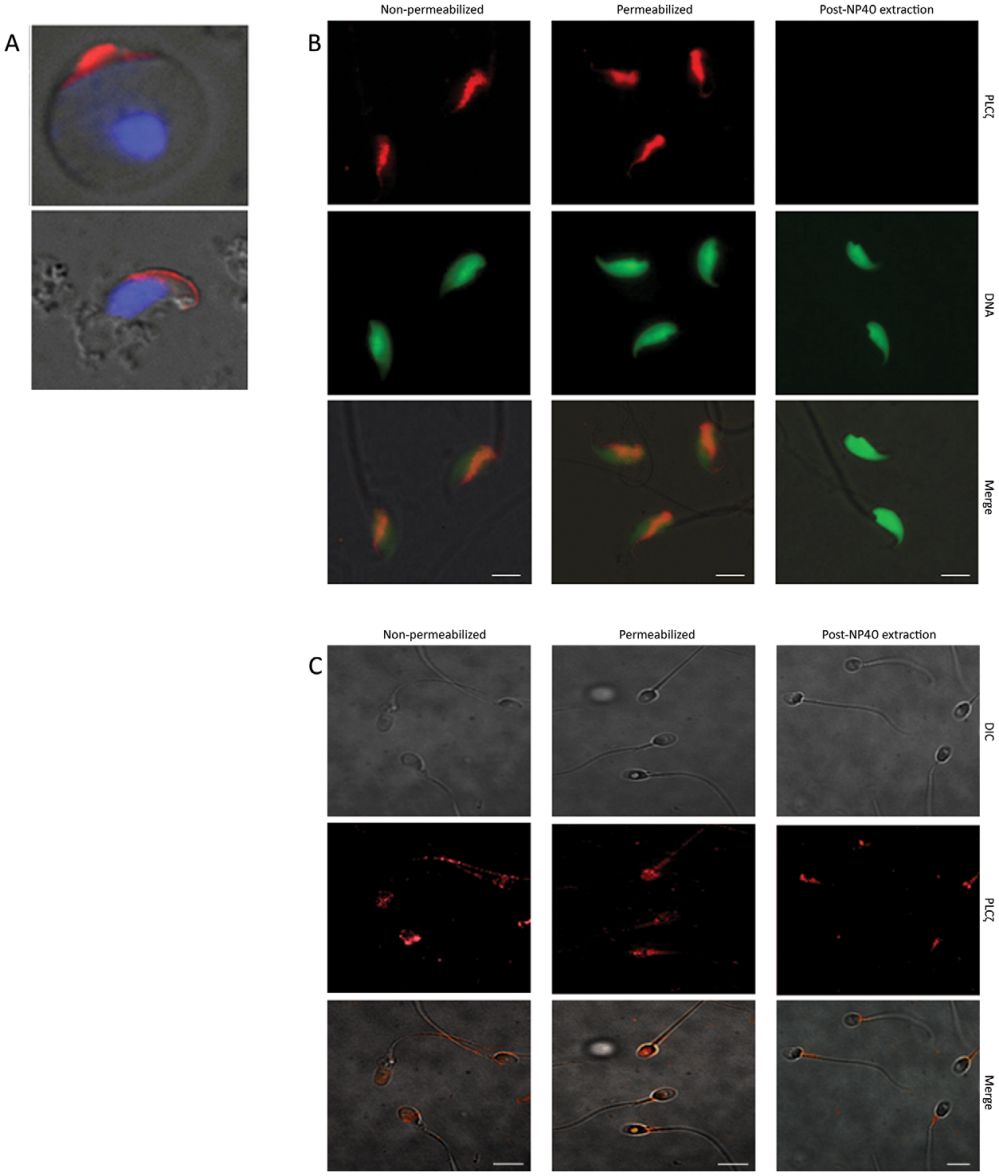
Localization of PLCζ in mouse testicular spermatids, and mouse and human mature spermatozoa by immunofluorescence. (**A**) PLCζ (red) localized to the forming acrosome in round spermatid (top) and elongated spermatid (bottom) of testicular spreads. DNA was stained by DAPI (blue). (**B, C**) Mature spermatozoa were extracted from cauda epididymis and ejaculate in mouse and human, respectively. The PLCζ immunofluorescence was detected in both non-permeabilized and permeabilized mouse (B) and human (C) spermatozoa. NP40 extraction abolished the detected signal on the sperm head, confirming the presence of PLCζ on the surface of mature spermatozoa. Immunolocalization above was done with anti-EF with a similar result obtained with anti-hmPLCζ. DNA staining is with DAPI in A (blue) and SYTOX green in B (green), DIC; differential interference contrast. Bars = 5 µm.

Immunogold and immunoperoxidase developmental data demonstrating PLCζ's acrosomal origin were reinforced by immunofluorescence performed on testicular germ cell spreads. As shown in Fig. 4A, PLCζ immunofluorescence was exclusive to the acrosome of mouse round and elongating spermatids. The predominance of PLCζ in the acrosome of spermatids suggested that the detergent soluble PLCζ-reactive band(s) detected in westerns of spermatozoa (Fig. 1) were most likely of acrosomal origin. However, the predominant immunofluorescence labelling of PLCζ in mouse cauda-epididymal spermatozoa was over the postacrosomal region of the sperm head (Fig. 4B). In humans, PLCζ also appeared to be localized over both postacrosomal and acrosomal regions (Fig. 4C). Because of this unexpected localization pattern after epididymal passage, the possibility of epididymal secretion and sperm surface acquisition of PLCζ was investigated. Comparing permeabilized *vs.* non-permeabilized spermatozoa, the immunofluorescence was similar suggesting localization of PLCζ to the sperm surface. Furthermore, removal of the plasma membrane-bound proteins and exposure of PAS-PT by NP40 eliminated PLCζ fluorescence. Complementary to our findings in mouse and human, PLCζ immunofluorescence in bull spermatozoa was localized to the postacrosomal region and was removable by NP40 extraction prior to fixation (Fig. S2).

### Expression of PLCζ in mouse epididymis

**Figure 5:**
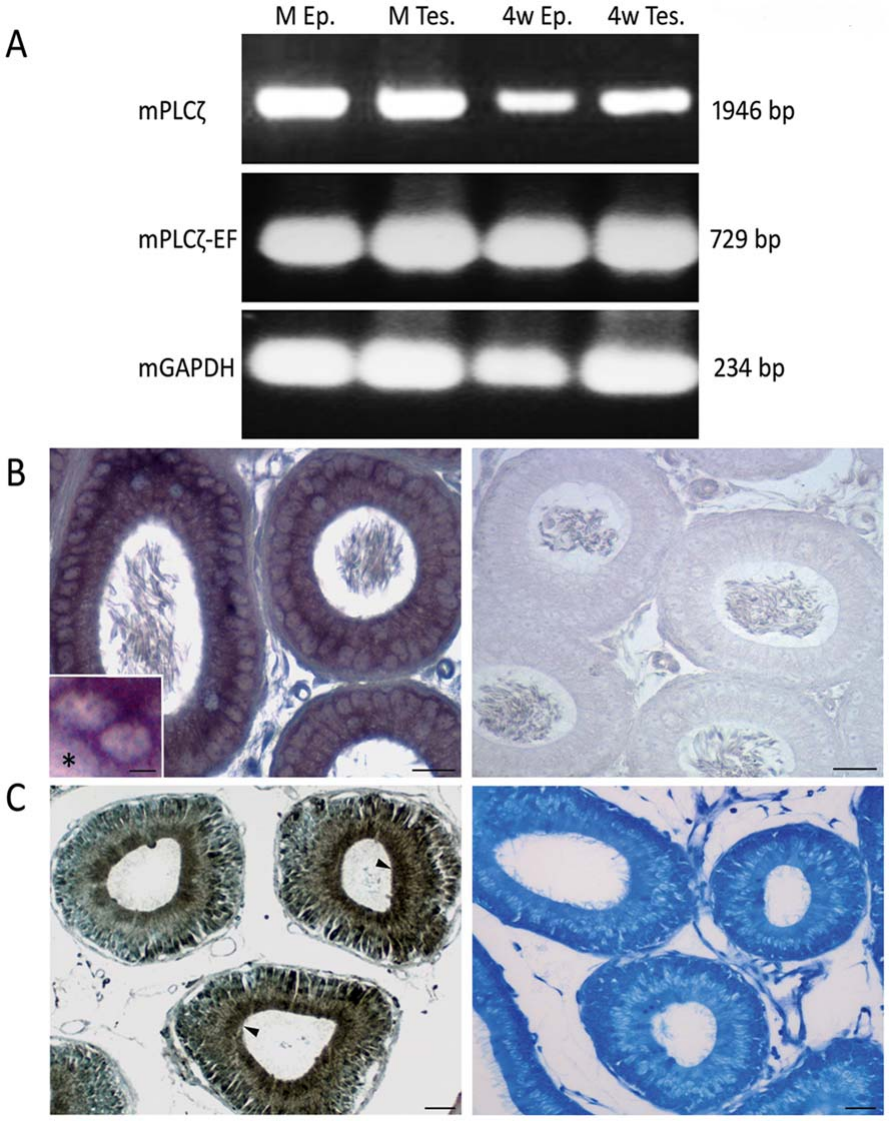
PLCζ mRNA and protein expression in mouse testis and epididymis. (**A**) RT-PCR detection of PLCζ mRNA (mPLCζ) in mature testis (M Tes.) and epididymis (M Ep.) of an adult mouse. mPLCζ was also detected in the 4 week old mouse epididymis (4w Ep.) excluding the possible contamination with RNA from testicular sources (4w Tes.). Primers were specifically designed to detect the full size open reading frame of mPLCζ. Utilizing second nested PCR, the results were confirmed by primers designed to detect the sequence encoding PLCζ EF hand domain (mPLCζ-EF). Mouse GAPDH was used as the house keeping gene for optimization of PCR results. (**B**) In situ hybridization of PLCζ mRNA on paraffin embedded mouse epididymal tissue. Inset shows perinuclear localization of mRNA within the principal cell. The interstitium within the inset is marked by asterisk. No signal is detected in the corresponding regions of control probe (right panel). (**C**) Paraffin embedded mouse epididymal tissues immunoproxidase stained with anti-EF antibody. Similar results were obtained by anti-hmPLCζ antibody. High level of PLCζ expression was detected in the apical region (arrowheads) of principal cells, where secretory vesicles accumulate and the supranuclear region of Golgi apparatus. Right panel shows the result of pre-incubating the primary antibody with the oligopeptides used to raise the immune serum. Bars = 25 µm Bar in the inset: 5 µm.

The finding that PLCζ localized to the cauda sperm head surface led to a hypothesis that PLCζ is not a strictly testis specific molecule as dogma would suggest, but also an epididymal secretory protein. To test this hypothesis, semi-quantitave reverse transcriptase polymerase chain reaction (RT-PCR), using specific PLCζ primers, was performed on total mRNA isolated from mature mouse testes and epididymides, revealing PLCζ mRNAs of similar size in both tissues. To avoid contamination of epididymal samples with testicular spermatozoa, tissues from 28 day old mice were also tested, providing a similar result to that found in adult tissue and confirming the epididymal source of PLCζ mRNA in mouse (Fig. 5A). Furthermore, we performed in situ hybridization to localize the mRNA in the epididymal epithelium. Our results confirmed the transcription of PLCζ mRNA in the principal cell of the epididymal epithelium and mostly in initial segment (not shown) and caput epididymis (Fig. 5B).

In order to provide evidence that PLCζ mRNA is translated in the epididymis, immunocytochemistry was performed on paraffin embedded mouse epididymal tissue. PLCζ immunostaining was detected in the principal cells of the epididymis, with the reaction product being most concentrated in the apical and supranuclear regions indicating Golgi apparatus contribution (Fig. 5C). The principal cells within initial segment of the caput epididymis were found to be most immunoreactive. Within the epididymal lumen, immunostaining was most evident in sperm tail and cytoplasmic droplet (not shown).

### PLCζ depletion after acrosome reaction during porcine *in vitro* fertilization

**Figure 6:**
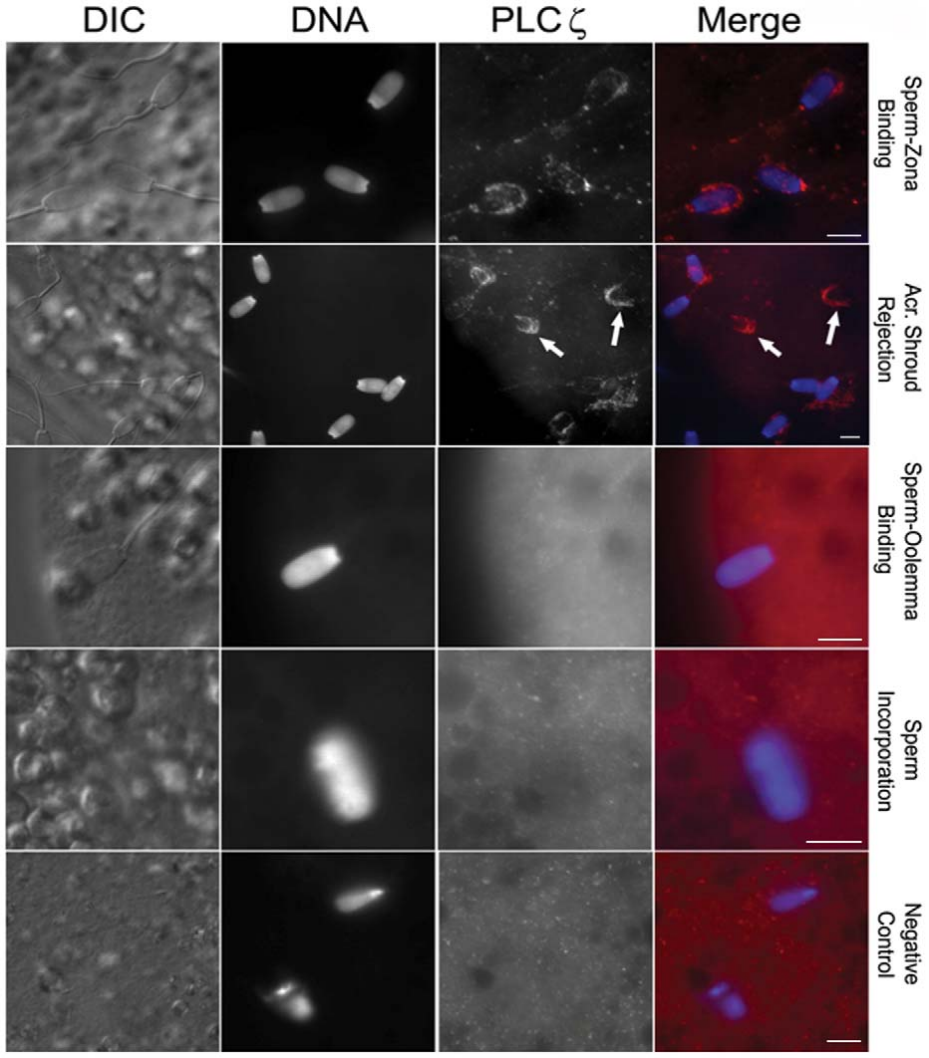
Indirect immunofluorescence analysis of PLCζ during *in vitro* fertilization in swine. Note the PLCζ immunoreactivity (red) in the acrosome region during sperm-zona binding. The immunoreactivity disappears along with acrosome shroud (arrows) when the spermatozoa undergo the acrosome reaction. No PLCζ immunoreactivity was detectable on the sperm heads during sperm-oolemma binding and incorporation into the oocyte. The results shown here are from anti-pPLCζ while a similar result was observed with anti-EF. DAPI (blue) was used for DNA staining; DIC, Differential Interference Contrast. Bars = 25 µm.

To answer whether the localization of PLCζ on the sperm head is appropriate for its suggested role as SOAF, the fate of boar sperm PLCζ during *in vitro* fertilization was followed. At the time of sperm-zona pellucida contact, PLCζ immunofluorescence predominated in the acrosomal region (Fig. 6). Subsequently, during the zona-induced acrosome reaction, the immunoreactivity accompanied the acrosomic shroud and was lost from the sperm head. After zona penetration and during sperm-oolemma binding and sperm incorporation into the oocyte cytoplasm, the PLCζ signal was no longer detectable (Fig. 6).

## Discussion

During fertilization, motile and fertilization competent spermatozoa pass through the female reproductive tract, interact with the zona pellucida and activate the metaphase II arrested oocytes after sperm-oolemma fusion [34]. The ability of spermatozoa to activate oocytes depends upon the normal expression of the sperm borne oocyte activating factor (SOAF) during spermiogenesis and its appropriate assembly as part of the PAS-PT [9], [10]. Failure of oocyte activation is considered to be responsible for 2–3% of failed fertilization cases when ICSI is performed [35]. Therefore, it is crucial to identify SOAF and its potential contribution to infertility, as well as to harness it for infertility treatments. Among the several SOAF candidates, PLCζ has been discussed most extensively in the last decade because its cRNA or recombinant protein induces calcium oscillations and oocyte activation when injected into the oocyte [19], [36]. Although there is much information about the characteristics of PLCζ such as its active domains [37] or expression in different species [18], little is known about its developmental expression during spermiogenesis, and localization in the spermatozoa before and during fertilization process.

### PLCζ's location is not consistent in spermatozoa between species

The importance of SOAF localization to the PT was highlighted when Kimura *et al.* [38] showed that the incubation of the sperm heads with non-ionic detergent, Triton X-100, removed all sperm membranous and acrosomal components, but left the PT and the oocyte-activating ability of the sperm head intact. The mandatory localization of SOAF to the PT was later confirmed by Perry *et al.* [39] and Sutovsky *et al.* [40]. In addition, the latter investigators provided convincing evidence that SOAF was regionalized to the PAS-PT, which is the first part of the PT solubilized on sperm entry into the oocyte cytoplasm. Therefore on the premise that SOAF should not be extractable by non-ionic detergents and should reside in the PT, we were perplexed to find that a 74 kDa protein corresponding to the catalytically active PLCζ isoform was extractable with non-ionic detergent from human spermatozoa, while a smaller, presumably inactive PLCζ isoform remained in the pellet. Grasa *et al.* [32] found a similar 74 kDa PLCζ band along with other smaller size bands in whole human sperm, while only the 74 kDa band was released to the sperm extract by freeze thaw, which is in agreement with our results.

In the mouse, on the other hand, the presumably full size active PLCζ isoform was detergent resistant. It was not retained in the sperm head as expected but rather in the sperm tail. Young *et al.* [30] showed a similar pattern of PLCζ immunoblotting in mouse where a 58 kDa band was extracted by Triton X-100, while the remaining PLCζ-reactive bands including the presumed active PLCζ band remained in the pellet [30]. However, they didn't further analyze whether the 74 kDa band belonged to the sperm head or tail. Similar to our findings in mouse, Bedford-Guaus *et al.* [28] immunolabelled the catalytically active PLCζ isoform in equine sperm tails. The sperm heads and tails were separated by sonication and centrifugation in this study, while no detergent extraction was performed. Overall, our findings show that the active, full size ∼74 kDa PLCζ isoform is localized to sperm fractions other than PT.

We detected smaller size PLCζ-reactive bands (50–60 kDa) which have been observed in other studies and often described as breakdown products, unrelated cross-reactive proteins [22], [28], [30], [32] or splicing variants [36]. Kurokawa *et al.* performed FPLC fractionation of porcine sperm extracts and, remarkably, detected immunoreactive full size PLCζ in a Ca^2+^ inactive fraction, while several fractions capable of inducing oscillations were devoid of functional PLCζ [41]. An alternative explanation for the bands below 74 kDa is that they represent other PLCζ isoforms. An isoform of PLCζ (NYD-SP27) with a size of approximately 55 kDa has been described recently which is localized to the sperm head as well as human pancreas [42], [43], [44]. This isoform lacks the functionally important EF domains and is suggested to act as an inhibitor of PLC family kinases. NYD-SP27 is proposed to promote capacitation when it detaches from sperm head [44]. We recommend further characterization of the undersized PLCζ-reactive bands as they may shed light on roles of sperm PLCζ irrelevant to oocyte activation.

### PLCζ is secreted as a component of the acrosome during spermiogenesis

Developmentally, the assembly of sperm PAS-PT occurs in elongating spermatids by microtubular manchette transport, and is independent of acrosomal formation [45], [46], [47]. Therefore, to be considered part of the PAS-PT, a SOAF candidate should be shown to originate in elongating spermatids during spermiogenesis. Contrary to this expectation, our immunocytochemical study shows, for the first time, that human and mouse PLCζ is secreted by the Golgi complex within proacrosomic granules that later fuse to form the AV. Interestingly, PLCζ in human and mouse appears to diminish from sperm acrosome towards the end of spermiogenesis. It is unlikely that this diminution in PLCζ concentration was due to a hidden epitope, as the inner acrosomal membrane protein IAM38 [48], which was used as a control with an identical origin to PLCζ, remained intense during spermiogenesis (data not shown). The expression of PLCζ mRNA has been shown in different phases of spermatogenesis among different species [25], [26], [27], [30], [49]. To our knowledge, there is only one study, in equine testis that shows PLCζ translation in the round spermatids [28] and one in the mouse that is inconclusive as to where the protein originates because only one of 12 stages of the cycle of seminiferous epithelium is represented at very low magnification [50].

Besides our immunolocalization of PLCζ in the acrosome, our fractionation/immunoblotting data indicates a sperm mitochondrial source of PLCζ. A recent study (*Proceedings of 11^th^ International Symposium on Spermatology)* shows that *Plcζ (−/−)* knockout mice demonstrate arrest of spermiogenesis at the level of round spermatids [51], a time period during spermiogenesis, according to our analysis, when PLCζ levels are highest. We recommend that consideration be given to PLCζ involvement in mitochondrial metabolism and acrosomal biogenesis during spermiogenesis.

### Epididymal cells secrete PLCζ which associates with the sperm head surface

We detected PLCζ immunofluorescence mainly in the postacrosomal region of mouse spermatozoa. In human spermatozoa, we localized it to both acrosomal and postacrosomal regions. The localization and intensity of labeling was similar in both permeabilized and non-permeabilized spermatozoa, suggesting the binding of PLCζ to the sperm surface. In addition, non-ionic detergent extraction coincided with the disappearance of immunolabelling, establishing that PLCζ was not a constituent of the detergent resistant perinuclear theca fraction which harbors SOAF. In mouse, PLCζ was localized by immunofluorescence to the PAS-PT and acrosomal regions of cauda-epididymal spermatozoa [29], [30]. Using an identical antibody as Young *et al.* [30], we could not replicate their results, where PLCζ was detected in PAS-PT after mouse sperm extraction with non-ionic detergent Triton X-100. In human, PLCζ has been localized over acrosomal, equatorial and postacrosomal regions of the ejaculated sperm head [32]. In equine spermatozoa, it was localized to the acrosome, equatorial region, and in the connecting piece and the principal piece of the sperm tail [28]. Because the studies above did not compare the immunolocalization between permeabilized and non-permeabilized sperm they do not distinguish whether PLCζ is within these components or on the membrane surface covering them.

The PLCζ immunofluorescence findings prompted us to hypothesize the epididymal expression of this sperm protein. We performed RT-PCR, in situ hybridization and immunocytochemistry to analyze PLCζ expression at the mRNA and protein levels, respectively. We demonstrated high level of mRNA expression and protein secretion in the mouse epididymis, both in adult and prepubertal males. Recent studies show PLCζ expression in mouse brain [52] as well as the puffer fish ovary and brain [53]. To our knowledge, this is the first time that PLCζ protein is reported in the mouse epididymis. These findings negate the thesis of testis specific origin of PLCζ. We propose that the signal detected on the head region of mature spermatozoa is most probably from the epididymal sources of PLCζ. This is an important consideration because the sperm acrosome and surface proteins are removed early during the fertilization, *i.e.* prior to sperm-oolemma fusion and sperm incorporation into the ooplasm, at which time the intact sperm PT becomes the only possible source of SOAF for inducing calcium oscillation and oocyte activation [9], [38], [40].

### Spermatozoon lacks PLCζ when it fuses with oolemma and enters ooplasm

Using established porcine IVF model, we demonstrated the pattern of PLCζ localization during *in vitro* fertilization for the first time and showed that PLCζ is mainly observed on the porcine sperm acrosomal region before fertilization. After the acrosome reaction, it is depleted and released together with acrosomal shroud. We were unable to detect any PLCζ in the postacrosomal sheath at an early stage of sperm incorporation, when its contents were dispersed into the ooplasm and activated oocyte. This is in stark contrast with well documented, easily traceable release of PAS-PT-resident signalling protein PAWP in the ooplasm during porcine fertilization [14]. Fertilization biology will benefit from further research aimed to understand PLCζ's role in the acrosome reaction during fertilization. Another critical step towards scrutinizing the currently proposed role of PLCζ in oocyte activation is to block the ‘sperm’ induced calcium oscillations during *in vitro* fertilization and after ICSI by PLCζ relevant antibodies or competitive peptides. The importance of this step is highlighted by the fact that the *Plcζ (−/−)* knockout mice are not appropriate models to study because of the arrest of spermiogenesis for unknown reasons [51]. As shown in the case of other SOAF candidate, PAWP, competitive peptides and antibodies were able to block sperm-induced intracellular calcium release and meiotic resumption in amphibians and mammalian oocyte activation, respectively, providing proof that PAWP has a non-redundant role in oocyte activation [14], [15].

Identification of SOAF could improve the understanding of the etiology of male infertility and failed fertilization after ART. SOAF could be used as a biomarker for accurate diagnosis of male infertility in couples with unexplained, idiopathic infertility, which comprises up to 20% of all infertility cases [54], [55], [56]. Some patients with globozoospermia have demonstrated defective or absent PLCζ in sperm extracts [22], [24]. However, as many other acrosomal and PT proteins are absent in these patients [57], there is no conclusive evidence showing that PLCζ is the actual cause of failed fertilization and infertility in these patients. The other important aspect of SOAF is highlighted in couples with low fertilization rate following ICSI, due to sperm failure to activate the oocyte. The availability of an authentic sperm borne oocyte-activating factor in recombinant form could enhance the efficiency of these treatments, at the same time being more efficient and safer than artificial oocyte activation by chemical methods or mRNA/cRNA injection [58]. The proper developmental analysis is required to make the scientific decision of whether a candidate protein is a SOAF. We strongly recommend the precise SOAF characterization as a prerequisite to determining SOAF candidates and finding the actual role of PLCζ in reproduction.

In conclusion, the present study shows that PLCζ is an intra-acrosomal protein that incorporates into the forming acrosome during spermatid differentiation, but contrary to expectations for a SOAF candidate, is not incorporated into the assembling PAS-PT. We demonstrate, for the first time, the expression of PLCζ in mouse epididymis that may result in the acquisition onto sperm head surface. During IVF-induced acrosome reaction on the zona pellucida surface, PLCζ disappears and does not appear to be incorporated into the oocyte cytoplasm during fertilization. We propose the possibility of acrosomal, surface and mitochondrial PLCζ compartmentalization in the spermatozoa. Our results along with the recent findings from other researchers make it unlikely that this member of phospholipase family is a genuine sperm-borne oocyte activating factor.

## Materials and Methods

All animal works in this study have been conducted according to national guidelines, approved by Queen's University Anima Care Committee (approval# Oko-2007-007-R3). Ethical approval for research on the human samples in this study was obtained from Queen's University Health Sciences Research Ethics Board. Written informed consents were obtained from all participants.

### Antibodies

To scrutinize its subcellular localization in spermatozoa, antibodies were raised against different regions of PLCζ, including novel target epitopes as well as epitopes targeted by antibodies in studies by other researchers. We raised affinity purified polyclonal rabbit anti-serum against the synthetic sequence comprising the EF hands as crucial domains for the function of PLCζ [59] in human, mouse and pig (C-KDNDRLKQGRITIEEF-coNH_2_), named as anti-EF in the text. The second affinity purified polyclonal rabbit anti-serum was raised following the procedures described by Young *et al.* [30] against two synthetic peptides of hamster, human and mouse PLCζ (C-MEMRWFLSKIQDEFRGGKI-coNH_2_ and C-CMNKGYRRVPLFSK-coNH_2_), named as anti-hmPLCζ. Additionally for human spermatozoa, we used the polyclonal rabbit antiserum against two peptides of human PLCζ (C-RESKSYFNPSNIKE-coNH_2_; C-ETHERKGSDKRGDN-coNH_2_), initially obtained from Dr. John Parrington, University of Oxford, and later purchased from Covalab (Villeurbanne, France), named as anti-hPLCζ. The immunoblotting and immunocytochemistry experiments were performed using different corresponding antibodies to achieve the most consistent results. In addition to anti-EF for porcine *in vitro* fertilization, we used polyclonal antiserum against 19-mer peptides of porcine PLCζ (C-MENKWFLSMVRDDFKGGK-coNH_2_), named as anti-pPLCζ, raised in rabbits following the procedures described by Kurokawa *et al.* [41].

### Sperm preparation and protein extraction

In order to localize the protein in the sperm compartments as accurately as possible, we chose to study different soluble and insoluble fractions of mouse and human sperm extracts. Sperm were obtained from the cauda epididymis of mature CD1 male mice (Charles River, St-Constant, QC, Canada). To extract the membranous components such as plasmalemma, acrosome and inner acrosomal membrane, the washed spermatozoa were treated with non-ionic detergent, 1% NP40 in the presence of 0.01 M phenyl methylsulphonyl fluoride (PMSF). To detach the sperm heads from the tails, the NP40 pellet was washed several times and sonicated with a Vibrocell Sonicator (50 Watt model, Sonics and Materials Inc., Danbury, CT) according to the previous protocol [60]. The efficiency of separation was monitored by phase contrast microscopy until >99% of head-tail junctions were broken. The head/tail mixture was further resuspended in buffer containing 80% sucrose. Following the previously described protocol [60], [61], sperm heads and tails were isolated on a sucrose gradient with over 99% purity confirmed by phase microscopy. Furthermore, isolated sperm tails were treated with Triton- dithiothretiol (DTT) mixture (2% Triton X-100, 5 mM DTT, 50 mM Tris-HCl, pH 9) in order to denude the mid-piece of the mitochondrial sheath [62].

Human spermatozoa were donated by three normal fertile males. The semen samples were allowed to liquefy, then washed and pooled together. The NP40 extraction was performed as described above. To mitigate the effects of repeated freeze-thaw process, all sperm samples were treated fresh and sperm fractions were used immediately kept in −80°C until further use.

### Immunoblotting

Sperm samples were dissolved in reducing sample buffer containing 2% SDS and 5% β-mercaptoethanol. Equal amounts of sperm extracts (1×10^7^ sperm/lane) were loaded and resolved on 10 or 12% SDS-PAGE. Proteins were transferred to either nitrocellulose (Schleicher and Schuell, Dassel, Germany) or polyvinylidene fluoride (PVDF; Millipore, Mississauga, ON) membranes. The immunoreactivity on western blots was detected with peroxidase labeled goat anti-rabbit IgG (Vector Laboratories, Inc., Burlingame, CA) diluted 1∶25,000 (v/v) and further use of enhanced chemiluminescent substrate (Pierce, Rockford, IL) with exposure to X-ray films.

### Immunoproxidase staining for light microscopy

The paraffin-embedded human testicular tissue, obtained from cancer patients who underwent total orchiectomy in Kingston General Hospital, Queen's University, along with mouse testicular and epididymal tissues were processed for immunoperoxidase staining according to previously described protocol [63]. Following deparaffinization in xylene and hydration through a concentration graded series of ethanol, slides were blocked with avidin- and biotin containing blocking serum followed by 10% normal goat serum [64], to avoid non-specific binding. Immunolabelling was conducted using an avidin-biotin peroxidase complex (ABC) kit (Vector Labs, Burlingame, CA). An antigen-retrieval technique was performed prior to primary antibody incubation to unmask the antigenic epitopes [46]. Slides were incubated with primary antibody overnight at 4°C, then washed in 25 mM Tris-buffered saline (TBS, pH 7.35) containing 0.1% Tween. Biotinylated goat anti-rabbit IgG was used as the secondary antibody (1∶200, Vector Labs, Burlingame, CA) for 1 h at room temperature, followed by incubation with ABC. Peroxidase reaction was visualized by incubation for 10 min with 0.03% hydrogen peroxide and 0.05% diamniobenzidine tetrachloride (DAB, Sigma, St. Louis, MO). The sections were counterstained after washing with 0.1% methylene blue, dehydrated in graded ethanol, immersed in xylene, and mounted in Permount, then visualized by Nikon Eclipse E800 microscope.

### Immunogold labelling and electron microscopy

Mouse testes were fixed in 4% formaldehyde and 0.8% gluteraldehyde and embedded in LR white (Polysciences, Inc., Warrington, PA) according to procedure described previously [46]. Tissue was then cut in ultrathin sections and mounted on Formvar-coated nickel grids (Polyscience, Inc., Warrington, PA). After blocking with 10% normal goat serum, the sections were incubated overnight at 4°C with primary antibody. Following washing, the sections were incubated with goat anti-rabbit IgG conjugated to 10 nm colloidal gold (1/20; Sigma, Mississauga, ON, Canada), and counterstained with uranyl acetate and lead citrate. The labelled sections were examined under a transmission electron microscope (Hitachi 7000).

### Immunofluorescence

Human and mouse spermatozoa were processed and mounted on coverslips according to the previous published methods [65], [66]. The sperm slides were immersed and fixed in 2% paraformaldehyde for 40 min at room temperature, then permeabilized in 0.1 M phosphate buffered saline (PBS) with 1% Triton X-100 for another 40 min at room temperature. A proportion of each sample was kept non-permeabilized to discriminate between the sperm surface and intracellular proteins. Additionally, to address whether observed patterns of immunostaining might be associated with protein localization to acrosomal and membranous structures, mouse and human samples were treated with 1% NP40 for 1 hour before fixation and processed for immunofluorescence as described above. After 25 min block in PBS- 0.1% tween containing 10% normal goat serum [64] at room temperature, the primary antibody was incubated overnight at 4°C, followed by 3× sequential washing in the labeling buffer. The second antibody mixture used to develop fluorescent reaction was TRITC-conjugated goat anti rabbit IgG (1∶80 in PBST with 1%NGS; Zymed Laboratories Inc., South San Francisco, CA). Either the DNA stains SYTOX Green (0.5 mM, Invitrogen, Burlington, ON) or DAPI (1∶80, Invitrogen, Burlington, ON) were mixed with the secondary antibody. The slides were mounted in an anti-fade mounting medium (VecaShield; Vector Labs, Burlingame, CA) and viewed by Zeiss AXIO Imager.A1 microscope with high numerical aperture objectives, and an AxioCam HRc camera operated by AxioVs40 V4.7 software (all Zeiss, Jena, Germany).

### Reverse-transcriptase PCR

The mRNA expression of PLCζ was investigated by reverse transcriptase polymerase chain reaction (RT-PCR) of the mouse epididymal and testicular tissues. The RNA was extracted using Trizol as described before [67], [68]. The epididymal lumen was washed with PBS several times to remove any possible contamination with testicular RNA. Synthesis of cDNA and semi-quantitative RT-PCR were accomplished with specific primers targeting mouse PLCζ using OneStep RT-PCR kit (Qiagen, Mississauga, ON) according to the manufacturer's protocol with minor modifications. Reverse transcription performed in 45°C/30 min followed by initial PCR activation step in 95°C/15 min. For first PCR, the primer sequences were 5′-CAAGCGGCCCAGATCATG-3′ and 5′-GCGTCAGTTACATGCGTCAC-3′, amplifying a 1946-bp product through 40 cycles of denaturation at 94°C/10 sec, annealing at 53°C/1 min and extension at 68°C/2 min, followed by final extension at 68°C/10 min. Second PCR was performed on this product using specific primers 5′-CGCAGAAGCAAGATGGTTTTT-3′ and 5′-TGGGTAATCAGATGTCACAAAGG-3′ amplifying a 729 bp product through denaturation at 94°C/3 min followed by 40 cycles of denaturation at 94°C/1 min, annealing at 51°C/1 min, extension at 72°C/1 min and final extension at 72°C/10 min. Mouse glyceraldehyde-3-phosphate dehydrogenase testis-specific isoform (GAPDHS) was used as the housekeeping sperm specific gene [69]. The primers used for this purpose were 5′-GGTCGGTGTGAACGGATTTGGC-3′ and 5′-GTGGGGTCTCGCTCCTGGAAGA-3′ amplifying a 234 bp product through denaturation at 94°C/3 min followed by 30 cycles of denaturation at 94°C/30 sec, annealing at 56°C/30 sec and extension at 72°C/40 sec and final extension of 72°C/10 min. All thermal cycling procedures were performed using PTC-100 thermal cycler (MJ Research, Watertown, MA). The PCR products were separated on a 1.5% agarose gel, stained with ethidium bromide, and visualized under UV light.

### *In situ* hybridization

Mouse PLCζ was obtained and amplified from mouse testis by RT-PCR as mentioned above. The primers were 5′-GAATTCCATGGAAAGCCAACTT-3′ and 5′-GCGGCCGCTCTGAAGTACCAAACATA-3′ amplifying 1939 bp product through denaturation at 94°C/3 min followed by 35 cycles of denaturation at 94°C/1 min, annealing at 55°C/1 min, extension at 72°C/2 min and final extension at 72°C/10 min. The cDNA was inserted into pET21b vector (Novagen, Canada) within the underlined restriction sites for EcoRI and NotI, respectively. The clone was sequenced (Eurofins MWG Operon, ON, Canada) and used as a template to amplify the approximately 300 bp product used for RNA labelling probe. The PCR was performed with 5′-TTACCCTCACAATCCGAATCATC-3′ and 5′-TAATACGACTCACTATAGGGGTTGGAAAACAGAGGAACACGA-3′ primers through denaturation at 94°C/3 min followed by 30 cycles of denaturation at 94°C/1 min, annealing at 56°C/1 min, extension at 72°C/30 sec and final extension at 72°C/10 min. The product was purified using QIAGEN Purification Kit (Qiagen, Mississauga, ON), then served as a DNA template to synthesize DIG-labeled probe using T7 RNA polymerase and digoxigenin-11-uridine-5′-triphosphate (Fermentas, Burlington, ON, Canada) according to manufacturer instructions with minor modifications.

CD1 mouse epididymal tissue was fixed in 4% paraformaldehyde, embedded in paraffin, sectioned and mounted onto poly L-lysine coated glass slides for in situ hybridization. The sections were dewaxed, rehydrated, permeabilized with proteinase K (20 µg/ml dissolved in TE: 100 mM Tris-HCl pH 7.5, 50 mM EDTA in DEPC treated water) at 37°C for 30 min, washed with PBS for 5 min, refixed with 4% paraformaldehyde for 10 min and washed twice with RNase free 2× saline-sodium citrate (SSC) buffer (300 mM NaCl, 30 mM sodium citrate, pH 7.2 in DEPC treated water; Sigma, Mississauga, ON, Canada) at 37°C for 5 min. The prehybridization performed by incubating the sections at 45°C for 60 min in the buffer containing 4× SSC, 10% dextran sulfate, 1× Denhardt's solution (0.02% of Ficoll® 400, polyvinyl pyrolidone and bovine serum albumin), 2 mM EDTA, 50% deionized formamide, salmon sperm DNA (500 µg/ml); all purchased from Sigma, Mississauga. ON, Canada. Hybridization solution containing 100 ng/ml of digoxigenin-labeled PLCζ riboprobes in the prehybridization solution was applied to each section and incubated in a moist chamber at 45°C overnight. Following hybridization, sections were washed twice with 2× SSC at 37°C for 5 min; four times with 60% formamide in 0.2% SSC at 37°C for 5 min and twice with 2× SSC at 37°C for 5 min. The sections were then blocked with 1% Blocking Reagent (Roche Diagnostics, Laval, QC, Canada) in 100 mM Tris-HCl pH 7.5, 150 mM NaCl at room temperature for 30 min, followed by incubation at room temperature for 3 h with 1∶500 of an alkaline phosphatase-conjugated anti-digoxigenin antibody (Anti-Digoxigenin-AP, Fab fragments, Roche Diagnostics, Laval, QC, Canada) diluted in the blocking solution. The slides were immersed in 100 mM Tris-HCl pH 7.5, 150 mM NaCl for 5 min and 100 mM Tris-HCl pH 9.5, 150 mM NaCl, 50 mM MgCl_2_, 10% polyvinyl alcohol (PVA) for 10 min. Sections were then immunostained at room temperature for 2 h in solution containing 34 mg/ml 4-nitroblue tetrazolium chloride (NBT), 18 mg/ml 5-bromo-4-chloro-3-indolyl-phosphate (BCIP) and 240 µg/ml levamisole (Sigma, Mississauga. ON, Canada) in 100 mM Tris-HCl pH 9.5, 150 mM NaCl, 50 mM MgCl_2_, 10% PVA. The sections were finally washed with TE buffer for 5 min, mounted in the mounting medium (VecaShield; Vector Labs, Burlingame, CA) and viewed by Zeiss AXIO Imager.A1 microscope with high numerical aperture objectives, and an AxioCam HRc camera operated by AxioVs40 V4.7 software (all Zeiss, Jena, Germany).

### Porcine Oocyte Collection and *in Vitro* Fertilization

Pre-pubertal gilts from a local slaughterhouse were used for collection of ovaries and oocytes as described previously [70]. Oocytes were washed and transferred into TCM-199 medium containing 10 ng/ml epidermal growth factor, 0.5 µg/ml FSH, 0.5 µg/ml LH, 0.1% polyvinyl alcohol and 0.57 mM cysteine (mTCM-199) at 39°C in 5% CO_2_ atmosphere in humidified air. The oocyte-cumulus cell complexes were transferred to mTCM-199 without FSH and LH after 22–24 h, followed by additional culture for 20 h under the same condition. Freshly ejaculated sperm-rich fraction was collected from a fertile boar as described previously [71]. Spermatozoa were re-suspended in modified Tris-buffered medium. IVF was performed with the final sperm concentration of 1×10^6^ cells/ml [70]. Fertilized oocytes were then cultured at 39°C in North Carolina State University (NCSU)-23 medium supplemented with 0.4% BSA in 5% CO_2_ atmosphere in humidified air. 6–8 h post-insemination, the oocytes were stripped of zona pellucida, fixed in 2% formaldehyde and permeabilized in 0.1% Triton X-100 to analyze the dynamics of PLCζ at different stages of fertilization [72]. Oocytes were sequentially incubated with the primary and secondary antibodies for immunofluorescence according to above mentioned protocol. The DNA stain DAPI (1∶80) was mixed with secondary antibodies [65].

## Supporting Information

Figure S1 **Control sections for** **Fig. 2** **where primary antibody was preincubated with oligopeptides used to raise the immune serum before incubation with tissue section.** Little to no immunostaining is present except a weak background staining in the interstitium, documenting the specificity of the primary antibody for the mouse (A) and human (B) spermatids. r, round spermatids. Bars = 20 µm.
(TIF)

Figure S2 **Localization of PLCζ in bull mature spermatozoa by immunofluorescence.** Bull spermatozoa were fixed and processed for immunofluorescence as described previously [66]. The PLCζ immunofluorescence was detected in post acrosomal region of permeabilized spermatozoa but not in NP40 extracted spermatozoa, similar to our findings in human and mouse. To confirm our results on NP40 extracted spermatozoa, we used anti-bull PAWP antibody as a control. PAWP is localized to sperm PAS and consequently is resistant to NP40. The results shown here are with anti-EF antibody. DNA staining was performed with DAPI, DIC; differential interference contrast. Bars = 5 µm.
(TIF)
