# Supplementary figures and images for: The Testicular and Epididymal Expression Profile of PLCζ in Mouse and Human Does Not Support Its Role as a Sperm-Borne Oocyte Activating Factor
Source: PLoS One. 2012 Mar 12;7(3):e33496. doi: 10.1371/journal.pone.0033496 (PMC3299792; doi:10.1371/journal.pone.0033496)

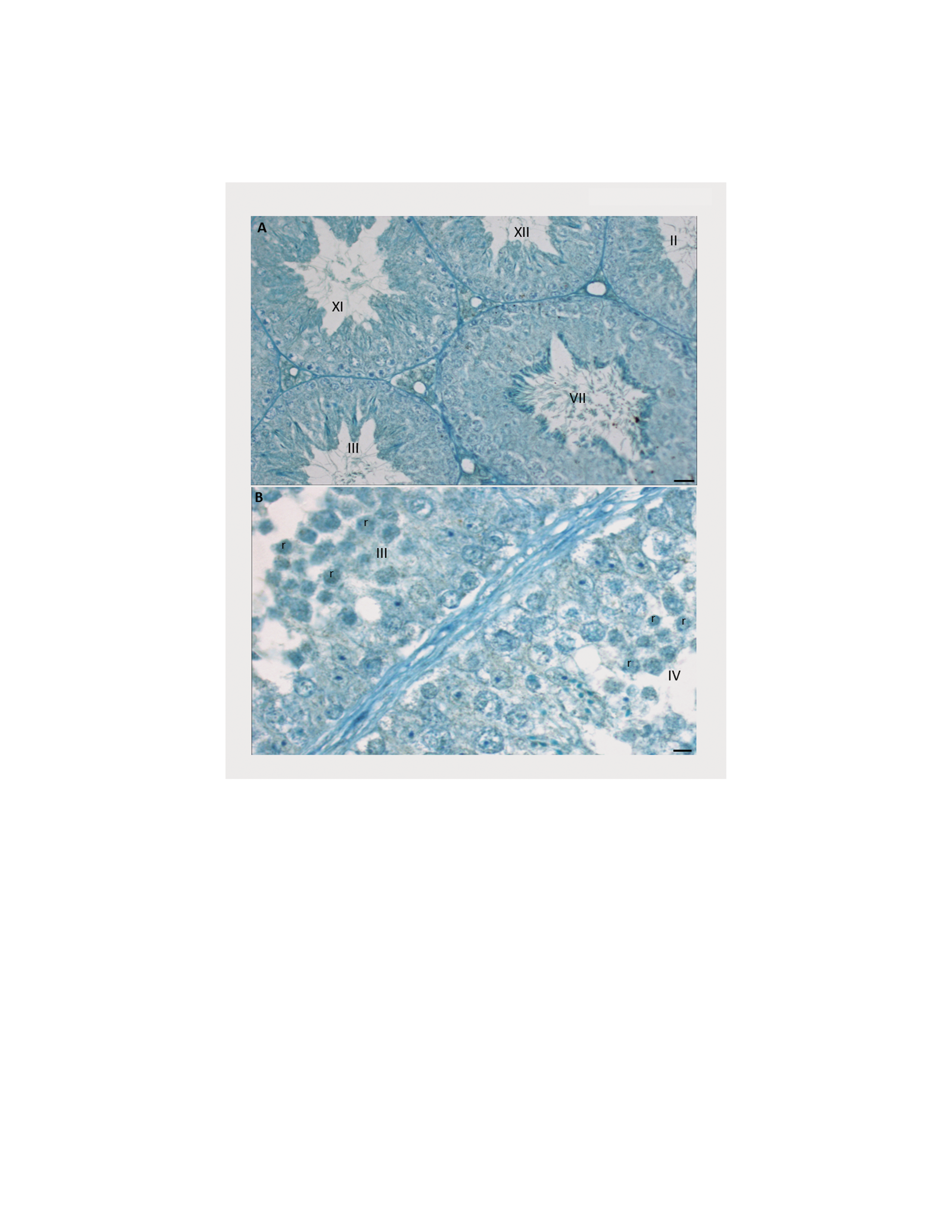

Supplement: Figure S1 — Control sections for Fig. 2 where primary antibody was preincubated with oligopeptides used to raise the immune serum before incubation with tissue section. Little to no immunostaining is present except a weak background staining in the interstitium, documenting the specificity of the primary antibody for the mouse (A) and human (B) spermatids. r, round spermatids. Bars = 20 µm. (TIF) [file pone.0033496.s001.tif]

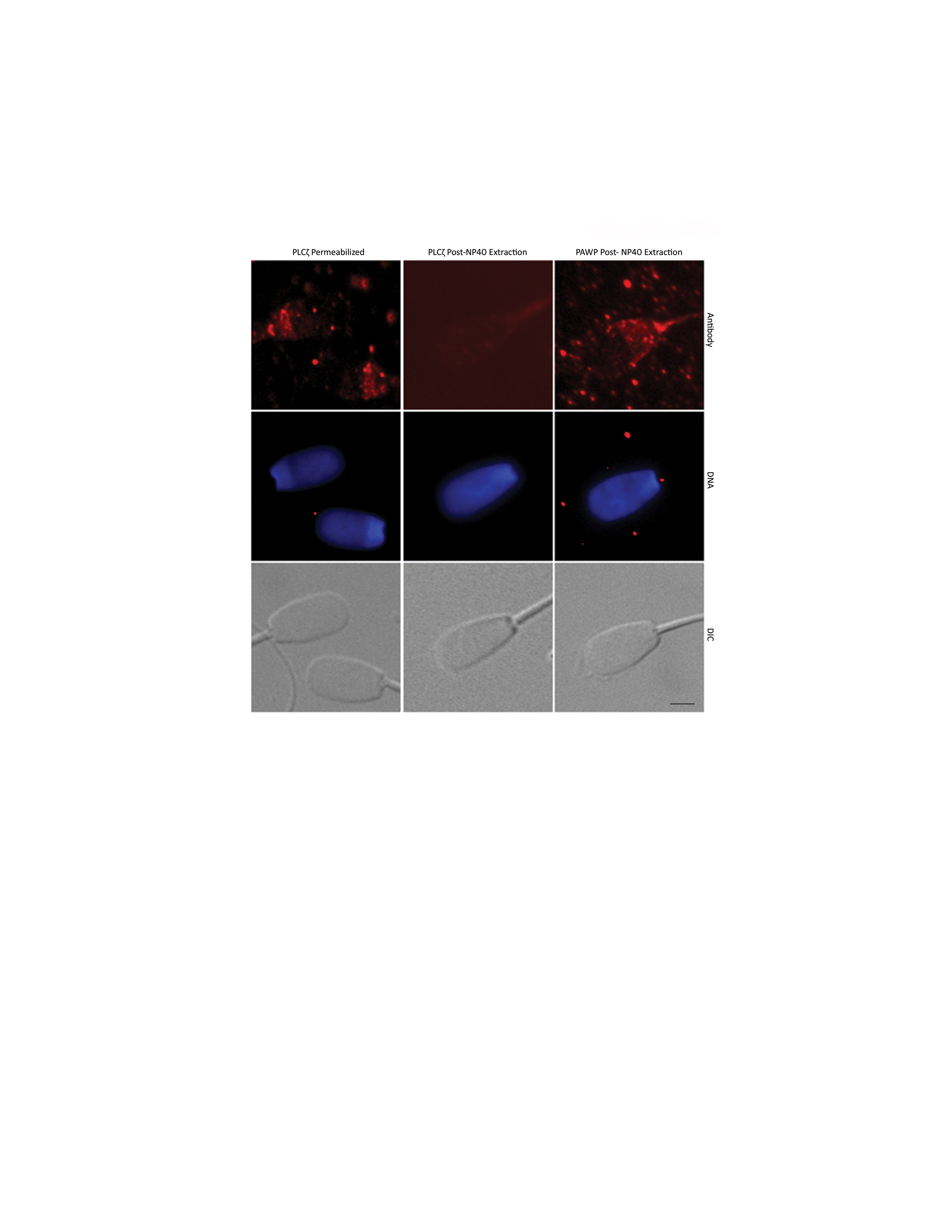

Supplement: Figure S2 — Localization of PLCζ in bull mature spermatozoa by immunofluorescence. Bull spermatozoa were fixed and processed for immunofluorescence as described previously [66]. The PLCζ immunofluorescence was detected in post acrosomal region of permeabilized spermatozoa but not in NP40 extracted spermatozoa, similar to our findings in human and mouse. To confirm our results on NP40 extracted spermatozoa, we used anti-bull PAWP antibody as a control. PAWP is localized to sperm PAS and consequently is resistant to NP40. The results shown here are with anti-EF antibody. DNA staining was performed with DAPI, DIC; differential interference contrast. Bars = 5 µm. (TIF) [file pone.0033496.s002.tif]
